# Inhibiting hepatocytes MAGL alleviates osteoporosis caused by high-fat diet-induced liver fibrosis in male mice

**DOI:** 10.1210/jendso/bvag114

**Published:** 2026-05-18

**Authors:** Qing Zhang, Zhixiang Lin, Peng Wang, Haixiang Xiao, Wenming Li, Lei Yu, Xiaolong Liang, Wentao Wang, Jing Qu, Gaoran Ge, Liangliang Wang, Hongtao Zhang

**Affiliations:** Department of Orthopedics, The Fourth Affiliated Hospital of Soochow University, Suzhou 215000, China; The Affiliated Huai’an Hospital of Xuzhou Medical University, Huai’an 223000, China; The First Affiliated Hospital of Soochow University, Suzhou 215000, China; The First Affiliated Hospital of Soochow University, Suzhou 215000, China; The First Affiliated Hospital of Soochow University, Suzhou 215000, China; The First Affiliated Hospital of Soochow University, Suzhou 215000, China; The First Affiliated Hospital of Soochow University, Suzhou 215000, China; Qilu Hospital of Shangdong University, Jinan 250100, China; The First Affiliated Hospital of Soochow University, Suzhou 215000, China; The First Affiliated Hospital of Soochow University, Suzhou 215000, China; Department of Cell Biology, Suzhou Medical College of Soochow University, Suzhou 215123, China; The First Affiliated Hospital of Soochow University, Suzhou 215000, China; Department of Orthopedics, The Second People's Hospital of Changzhou, The Third Affiliated Hospital of Nanjing Medical University, Changzhou 213000, China; Department of Orthopedics, The Fourth Affiliated Hospital of Soochow University, Suzhou 215000, China; The First Affiliated Hospital of Soochow University, Suzhou 215000, China

**Keywords:** hepatic osteoporosis, liver-bone axis, monoacylglycerol lipase

## Abstract

Hepatic osteoporosis is mediated through the liver–bone axis, but the precise mechanisms underlying osteoporosis in severe liver disease remain elusive. A high-fat diet can induce hepatic steatosis, which may subsequently progress to liver fibrosis. Monoacylglycerol lipase (MAGL), a rate-limiting enzyme in monoacylglycerol degradation, exhibits elevated expression in the liver. The potential of hepatic MAGL inhibition to attenuate osteoclast activation and mitigate osteoporosis remains unexplored. This study demonstrated that a high-fat culture medium upregulated MAGL and CB1R expression in mice hepatocytes. Pharmacological inhibition of MAGL using MJN110 resulted in reduced CB1R expression and decreased levels of the apoptosis marker SMAD3, indicating a reduction in liver fibrosis and hepatocyte apoptosis, respectively. Furthermore, we confirmed that CB1R expression decreased in mice hepatocytes with silenced mgll gene expression, mirroring the reduction in MAGL expression. Subsequent stimulation of osteoclasts with concentrated supernatants from hepatocytes cultured in control and mgll-silenced groups revealed a significant reduction in osteoclast activation markers via Western blot and PCR analyses. In vivo, mice fed a high-fat diet to induce liver fibrosis exhibited significant alleviation of liver fibrosis following intraperitoneal injection of MJN110. Notably, osteoclast activation in the femoral germinal layer of mice with liver fibrosis was elevated, whereas this activation was attenuated in the MJN110-treated group. In conclusion, these findings suggest that hepatic MAGL inhibition can alleviate femoral osteoclast activation and bone destruction associated with high-fat diet-induced liver fibrosis, offering a novel therapeutic avenue for hepatic osteoporosis.

The liver is a vital organ with multifaceted functions. It plays a crucial role in the metabolism of carbohydrates, lipids, and proteins, and also secretes cytokines and hormones [1] that influence distant organs. Clinically, patients with severe liver diseases, such as cholestatic liver disease and viral hepatitis, frequently exhibit osteoporosis. This condition, often linked to cholesterol transport [2] and other factors, is termed hepatic osteodystrophy [3]. Osteoporosis in other patient populations is associated with the systemic inflammatory cascade of proinflammatory cytokines induced by fatty liver [4]. In essence, osteoporosis secondary to liver disease is referred to as hepatic osteoporosis. Conversely, some studies suggest that nonalcoholic fatty liver disease (NAFLD) may have a protective effect on bone mineral density (BMD) in female patients, although the study could not differentiate between simple steatosis, steatohepatitis, and fibrosis [5]. Based on the analysis of multiple studies [6, 7], the author observed that the majority of patients or experimental animals with osteoporosis share a common disease process of liver fibrosis. The author posits that this may be related to the liver's robust compensatory capacity in the setting of fatty liver rather than liver fibrosis, thereby eliminating the cause of hepatic osteoporosis, which warrants further investigation. Osteoclast-mediated bone resorption is a key mechanism in the pathogenesis of osteoporosis. Monoacylglycerol acyltransferase 2 (MGAT2), an enzyme highly expressed in the human small intestine and liver, regulates triglyceride absorption and homeostasis and significantly ameliorates liver fibrosis induced by high-fat diets [8]. Therefore, based on these prior investigations, we hypothesize that liver fibrosis resulting from a high-fat diet may activate osteoclasts and enhance pathological bone resorption, ultimately leading to osteoporosis.

Monoacylglycerol lipase (MAGL), a prominent enzyme in the brain and liver, plays a crucial role in lipid metabolism. Initially isolated from adipose tissue in the 1970s, MAGL catalyzes the terminal enzymatic step in triglyceride degradation. This rate-limiting enzyme hydrolyzes monoesters into glycerol and fatty acids (FA), and converts 2-arachidonoylglycerol (2-AG) into arachidonic acid and glycerol. MAGL is implicated in the promotion of inflammation, potentially exacerbating hepatic inflammation and fibrosis [9]. Furthermore, it is associated with various central nervous system disorders, including neuroinflammation, cognitive impairment, epileptogenesis, nociception, and neurodegenerative diseases [10], and is also implicated in cancer pathogenesis [11]. Current research on hepatic osteoporosis suggests that its etiology is primarily attributed to inflammation and oxidative stress, metabolic dysregulation (including lipid and iron metabolism), and endocrine factors and key gene alterations [12]. Consequently, we hypothesize that MAGL, given its involvement in hepatic lipid metabolism and its capacity to promote liver inflammation and fibrosis, may be linked to the inflammatory mechanisms underlying hepatic osteoporosis.

The endocannabinoid system (ECS) was identified in the mid-20th century. It comprises cannabinoids, cannabinoid receptors type 1 and 2 (CB1R, CB2R), endogenous lipid ligands, and their synthesis and degradation pathways, impacting skin physiology, immune responses, metabolism, and neural functions. Animals lacking hepatic CB1R do not develop dyslipidemia when subjected to a high-fat diet, whereas high-fat diet or alcohol-induced hepatic steatosis correlates with elevated CB1R expression [13]. Normal hepatocytes express only CB1R and not CB2R, but human hepatocellular carcinoma cell lines express CB2R. Furthermore, CB1R expressed by hepatocytes and hepatic myofibroblasts contributes to steatosis, liver regeneration, and liver fibrosis induced by high-fat and alcohol consumption [14]. Consequently, CB1R serves as a significant observation index in the progression of hepatic steatosis and liver fibrosis.

This study demonstrated that high-fat diet-induced MAGL-mediated hepatic fibrosis promoted osteoclast activation within the murine femoral germinal layer, leading to bone resorption, whereas a MAGL inhibitor (MJN110) attenuated this process.

## Materials and methods

### Animals

Six-week-old male C57BL/6J mice were randomly assigned to 3 groups (*n* = 6). The HF (high-fat control) and HF + MJN110 (high-fat experimental) groups were fed a 60% high-fat diet (Nantong Teluofei Feed Technology Co., Ltd.) for 16 weeks to induce fatty liver and hepatic fibrosis, while the control group received standard chow for the same duration. Subsequently, mice in the high-fat experimental group received daily intraperitoneal injections of MJN110 (10 mg/kg, MJN110: DMSO: mineral oil (volume ratio) = 1:1:18, MJN110 (Medchem Express (MCE)), mineral oil (Shanghai Aladdin Biochemical Technology Co., Ltd. (mouse embryonic cell culture grade))) for 7 consecutive days. The remaining 2 groups received equivalent volumes of mineral oil based on body weight. All animals were maintained under standard conditions: a 12-hour light/dark cycle, a room temperature of (21 ± 2) °C, and a relative humidity of (60 ± 10)%. Seven days later, blood was collected via eyeballs enucleation under sodium pentobarbital anesthesia, and liver and femur samples were harvested for subsequent analyses. All animal procedures were approved by the Animal Ethics Committee of Soochow University (SUDA20221124A11) and conducted in strict accordance with the approved guidelines and regulations.

### Cell culture and treatment

AML12 murine hepatocytes were obtained from the Cell Bank of the Chinese Academy of Sciences and maintained in DMEM/F12 medium supplemented with 10% fetal bovine serum (FBS), ITS medium supplement, and dexamethasone (40 ng/mL). Cultures were maintained at 37 °C in the 5% CO_2_ atmosphere. Group C (control) cultures utilized 1% fatty acid-free bovine serum albumin in place of 10% FBS. Palmitic acid and oleic acid were solubilized in 75% ethanol, dissolved in a water bath at 55 °C, and mixed on an oscillator. The resulting solution was then filter-sterilized using a 0.22 μm filter and added to the Group C culture medium after cooling to 37 °C. The Group F (high-fat) culture medium was prepared by supplementing the medium with palmitic acid (250 μM) and oleic acid (500 μM). Group M cultures received Group C medium supplemented with MJN110 (100 nM, with DMSO as a cosolvent). Group FM cultures received Group F medium supplemented with MJN110 (100 nM). Subsequent experiments were performed following 72 hours of treatment. For experiments involving mgll gene silencing, further experiments were performed after 72 hours of culture in Group C medium.

### Lentivirus transfection

The shRNA lentiviruses targeting the silencing of the mgll gene in mice hepatocytes were obtained from Jima Gene. Preliminary experiments were performed according to the manufacturer's protocol. Briefly, the virus blank control group and the virus demonstrating the highest transfection and silencing efficiency determined by PCR among the 3 mgll gene-silencing viruses were transduced into 6-well plates of AML12 cells (2*10^5^/well) at a multiplicity of infection (MOI) of 100. Following 24 hours of transduction, the culture medium was replaced with fresh medium containing 1 μg/mL puromycin—a concentration confirmed by a 72-hour viability assay—to eliminate untransduced cells, with subsequent expansion and passaging of the cells. Upon confirmation of robust and stable cell viability, the subsequent experiment was initiated. In the mgll gene silencing experiment, AML12 hepatocytes were cultured in high-fat medium (Group C) for 72 hours prior to further experimentation. The experimental groups were designated as a blank virus group (FNC group) and an mgll gene-silenced group (FMgll-group). The supernatant from both hepatocyte groups was collected and recentrifuged at low speed to remove any floating cells, cellular debris, and other impurities. Subsequently, ultracentrifuge tubes (Millipore) with 10 K molecular weight cutoff, capable of retaining proteins based on the molecular weight of MAGL, were employed to concentrate the supernatant to approximately 100 μL. The culture mediums were then replaced and combined with the concentrated supernatant to stimulate a response in RAW264.7 cells that had been preinduced with osteoclasts. After 72 hours of culture, the cells were harvested for further analysis.

### Cell proliferation toxicity assay

Cell proliferation toxicity assays of AML12 and RAW264.7 cells, induced by receptor activator of nuclear factor kappa-B ligand (RANKL), were conducted using the CCK-8 method. A 100 μL aliquot of cell suspension was seeded into each well of a 96-well plate, with 5 replicates per concentration. Following a 24-hour incubation, CCK-8 reagent and culture medium were combined at a 1:9 ratio, and 100 μL was added to each well of the inoculated plate. The culture plate was then incubated for 1-4 hours, and absorbance was measured at 450 nm using an ELISA reader, so as to confirm the maximum dosing concentration that has no significant statistical significance (*P* ≥ .05) compared with the negative control.

### Western blotting

AML12 hepatocytes and RANKL-stimulated RAW264.7 cells were lysed in RIPA buffer supplemented with 1% protease and phosphatase inhibitors. Protein concentrations were subsequently determined using a BCA protein assay, and the total protein contents were normalized across all experimental groups. Equal protein aliquots were resolved via 10% and 12% SDS–PAGE and transferred to PVDF membranes. Following 1 hour blocking step with 5% skim milk at room temperature, membranes were probed with specific primary antibodies overnight at 4 °C, followed by incubation with appropriate secondary antibodies for 1 hour at room temperature. Immunoblots were visualized using an ultrasensitive ECL chemiluminescence detection reagent and imaged with a chemiluminescent imaging system (Bio-Rad). Band intensities were semiquantified using Image J software. The following antibodies were used for immunoblotting analysis: Rabbit anti-CB1R (ABclonal Cat#A1447, RRID: AB_2761341) primary antibody (1:750), Rabbit anti-β-actin (ABclonal Cat#AC026,RRID: AB_2768234) primary antibody (1:160000), Rabbit anti-MAGL (ABclonal Cat#A6654,RRID:AB_2767241) primary antibody (1:750), Rabbit anti-SMAD3 (ABclonal Cat#A16913,RRID:AB_2772307) primary antibody (1:750), Rabbit anti-NFATc1 (Proteintech Cat#66963-1-Ig,RRID: AB_2882286) primary antibody (1:5000), Rabbit anti-CTSK (ABclonal Cat#A1782,RRID:AB_2763824) primary antibody (1:250), Goat Anti-Rabbit (ABclonal Cat#AS014, RRID:AB_2769854) secondary antibody (1:5000).

### Real time PCR

Total RNA was extracted using Trizol reagent. cDNA synthesis was performed using a reverse transcription system. Real-time PCR was conducted on a Bio-Rad PCR instrument, and data were analyzed using the Bio-Rad CFX Manager 3.1 software. Relative gene expression was normalized to β-actin. The primer sequences are shown in Table 1.

**Table 1:** Primer sequences of genes

Genes	Forward (5′→3′)	Reverse (5′→3′)
Mmp9	CGACTTTTGTGGTCTTCCCCA	TAGCGGTACAAGTATGCCTCTGC
Nfatc1	GACCCGGAGTTCGACTTCG	TGACACTAGGGGACACATAACTG
Acp5 Actin	CACTCCCACCCTGAGATTTGT GGAGATTACTGCCCTGGCTCCTA	CATCGTCTGCACGGTTCTG GACTCATCGTACTCCTGCTTGCTG

### ALT concentration determination, trap staining, and tissue section pathology

Following retroorbital blood collection from the experimental mice, serum was isolated via centrifugation. Alanine aminotransferase (ALT) activity was assessed using a commercially available assay kit (Nanjing Jiancheng), adhering to the manufacturer's protocol, and quantified via a microplate reader at 505 nm absorbance. Osteoclast differentiation was induced and visualized using a Trap staining kit, per the manufacturer's instructions, and imaged via microscopy. Liver specimens underwent HE staining, Sirius red staining, and α-sma immunostaining, while femur samples were subjected to HE and Trap staining. All staining and sectioning were outsourced to Anhui Ketu Company. Except for α-sma immunostaining, which was performed by the company, all other experimental results were captured by the experimenter using a microscope.

### Analysis of bone mineral density in trabecular of mice distal femurs

Following euthanasia of the experimental mice, femoral specimens from each experimental group were harvested concurrently and subjected to formalin fixation. Subsequently, micro-CT scanning (Skyscan1176) was performed. Scanned images were reconstructed using the NRecon software and imported into the DataView software for femoral image positioning and analysis. Finally, the images were imported into CTan software. The growth bridge, identified at the elevated region of the growth plate within the DataView software, served as the reference layer. The starting point for femoral trabecular analysis was established at a 60-layer offset toward the femoral backbone. A 150-layer segment was selected in the femoral backbone direction. The average bone mineral density of the 150-layer femoral trabecular bone was then calculated using this software.

### Statistical analysis

All data were analyzed using GraphPad Prism 9 to generate histograms. Experimental data are presented as mean ± mean ± SEM. Each experiment included a minimum of 3 biological replicates. Statistical comparisons between 2 experimental groups were performed using a *t*-test, while one-way analysis of variance (ANOVA) followed by Turkey's multiple comparison test was used for multiple experimental groups. *P* value < .05 was considered statistically significant. Prior to assessing statistical significance, the homogeneity of variance between the comparison groups (control group vs each experimental group) was confirmed to ensure the appropriateness of the statistical analysis.

## Results

### High-fat diet-induced hepatic steatosis was associated with elevated MAGL expression

**Figure 1:**
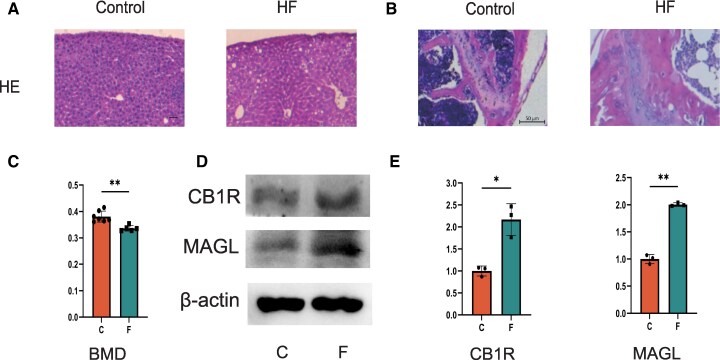
Liver damage in mice caused by high-fat diets leads to a decrease in the bone mineral density of femoral trabecular. A. Representative images of mouse liver HE staining. B. Representative images of HE staining of the lower femur of mice. C. Statistical analysis of bone mineral density in mouse femoral trabecular. D. Changes in CB1R and MAGL proteins in hepatocytes under blank control medium (C) and high-fat medium (F). E. Statistical analysis of protein grayscale in experimental group C using ImageJ. *P < .05, **P < .01. *t*-test (C, E). Data are represented as mean ± SEM.

To assess the efficacy of high-fat diet (HFD) modeling in inducing hepatic fibrosis in mice, we performed hematoxylin and eosin (HE) staining on liver sections from experimental animals. Histological analysis of HE-stained liver sections revealed the presence of numerous lipid droplets, indicative of steatosis, in the HFD group. Furthermore, the liver lobules exhibited architectural distortion, reflecting the ongoing cycles of hepatocyte injury and repair, consistent with the development of liver fibrosis (Fig. 1A). To determine whether HFD-induced liver fibrosis correlated with alterations in bone mass, we conducted HE staining on distal femur sections. The results showed that the femoral germ layer of the mice in the HF group had become thinner, and more cavity formed after the dissolution of fat could be seen in the bone marrow cavity (Fig. 1B). Subsequent computed tomography (CT) scans and statistical analysis of the femurs (Fig. 1C) demonstrated a significant reduction in trabecular bone mineral density in the HFD group (Group F) compared to the control group (Group C), indicating the development of osteoporosis.

We subsequently performed in vitro investigations to assess the impact of a high-fat culture medium on hepatocytes. Prior research has demonstrated that a high-fat culture medium can upregulate the expression of MAGL in hepatocytes [15], and MAGL is implicated in the pathogenesis of liver injury. To validate this observation, we utilized palmitic acid and oleic acid to formulate a high-fat culture medium within a serum-free medium, a method commonly employed to induce hepatic steatosis in hepatocytes. The final concentrations were established at palmitic acid (250 μM) and oleic acid (500 μM), and these were added to the serum-free culture medium following CCK8 assays, which confirmed the absence of significant effects on hepatocyte proliferation. Concurrently, we observed that CB1R, a key marker in the progression of hepatic steatosis and liver fibrosis, also increased (Fig. 1D), with statistically significant elevations in both MAGL and CB1R (Fig. 1E). These findings suggest that the formulated high-fat culture medium exerts detrimental effects on hepatocytes function.

### MJN110 intervention attenuated the detrimental effects of high-fat culture medium on hepatocytes

**Figure 2:**
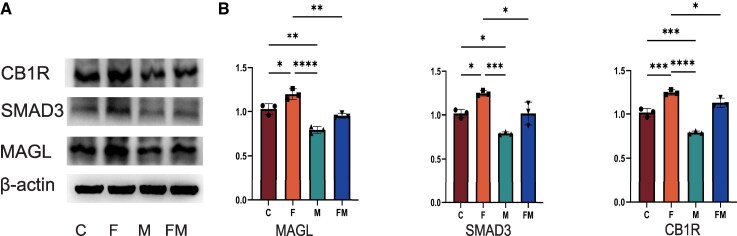
Effects of high-fat and MJN110 conditions on hepatocyte protein expression. A. Changes in MAGL, SMAD3, and CB1R proteins in hepatocytes under blank control medium (C), high-fat medium (F), blank medium with MJN110 (M), and high-fat medium with MJN110 (FM). B. Statistical analysis of protein grayscale in experimental group C using ImageJ. *P < .05, **P < .01, ***P < .001, ****P < .0001. Paired t-test (B). Data are represented as mean ± SEM.

To investigate the role of MAGL in high-fat medium-induced hepatocyte injury and elucidate the underlying mechanisms, we designed an intervention experiment utilizing MJN110 to inhibit MAGL protein synthesis. Following confirmation that 100 nM MJN110 did not significantly impact hepatocytes proliferation via CCK8 assays, we established the M group, treated with MJN110 (100 nM), and the FM group, treated with F medium supplemented with the same concentration of MJN110. The results demonstrated that MJN110 effectively inhibited MAGL protein synthesis, concurrently reducing CB1R protein synthesis. Furthermore, we incorporated SMAD3 detection as a specific marker of cellular apoptosis, confirming that MAGL-mediated hepatocyte damage influences the apoptotic pathway (Fig. 2A).

Notably, our experimental findings demonstrate that MJN110 effectively suppresses MAGL synthesis in both lipid-depleted and high-fat culture conditions. This inhibitory action resulted in a significant reduction in hepatocyte steatosis and the hepatic fibrosis marker CB1R, relative to the high-fat cultured hepatocyte group (F group). Concurrently, the critical hepatocyte apoptosis marker, SMAD3, exhibited a significant decrease (Fig. 2B).

### Following mgll gene silencing, the hepatocytes injury index was attenuated, which was accompanied by a reduction in the extent of osteoclast activation

**Figure 3:**
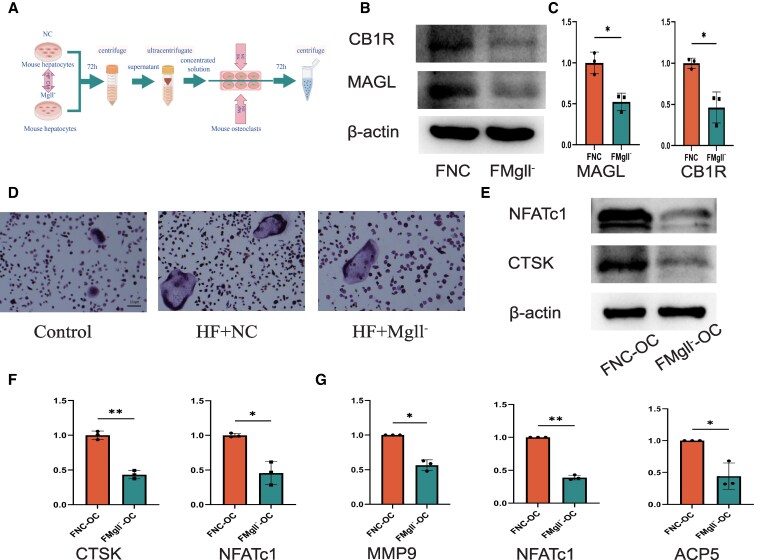
Experiment of hepatocytes after silencing the Mgll gene and coculture with osteoclast-induced raw cells. A. Schematic diagram of coculture of osteoclast-induced raw cells with NC group hepatocytes and Mgll-silenced hepatocytes supernatant under high-fat culture medium conditions. B. Protein expression of NC group and Mgll-silenced hepatocytes under high-fat culture medium conditions. C. Statistical analysis of protein grayscale was performed in experimental group B using ImageJ. D. Typical images of Trap staining of coculture of osteoclast-induced raw cells with hepatocytes supernatant in normal blank control group, HF + NC group, and HF + Mgll- group. E. Expression of osteoclast-specific proteins after coculture of osteoclast-induced raw cells with NC group hepatocytes and Mgll-silenced hepatocytes supernatant under high-fat culture medium conditions. F. Statistical analysis of protein grayscale in experimental group E using ImageJ. G. Statistical analysis was performed on the relative expression of osteoclast-specific genes after coculture of osteoclast-induced raw cells using the supernatant of NC group hepatocytes and Mgll genome-silenced hepatocytes under high-fat culture conditions.*P < .05, **P < .01. Paired *t*-test (C, F, G). Data are represented as mean ± SEM.

To ascertain whether the observed amelioration of hepatic function and the reduction in femoral osteoclast activation were directly attributable to the MAGL inhibitor MJN110, we conducted further investigations utilizing hepatocytes subjected to lentiviral-mediated silencing of the mgll gene, alongside a blank NC group lacking gene silencing (Fig. 3A). The experimental results demonstrated that, under high-fat medium conditions, the expression of MAGL in hepatocytes with silenced mgll gene was significantly diminished, accompanied by a marked reduction in CB1R, a representative marker of hepatocyte injury (Fig. 3B and C). Based on prior research, MAGL is localized within the plasma membrane, endoplasmic reticulum, and lipid droplets. We employed the extracted concentrated supernatant to stimulate Raw264.7 cells, which had been differentiated into osteoclasts. Considering the established presence of MAGL in the plasma membrane, endoplasmic reticulum, and lipid droplets [16, 17], and the potential for lipid droplets to mediate the extracellular release of MAGL via vesicles, a 10K ultracentrifuge was utilized to concentrate the supernatant, thereby preventing MAGL protein leakage. We selected CTSK and NFATc1, 2 pivotal proteins involved in osteoclast activation, for western blot analysis. Semiquantitative analysis using Image J revealed a significant reduction in the production of CTSK and NFATc1 proteins by hepatocytes with silenced mgll gene (Fig. 3E and F). Furthermore, we performed PCR assays on MMP9, NFATc1, and ACP5, which are key genes expressed during osteoclast activation. Semiquantitative analysis of expression levels corroborated the protein expression findings, demonstrating a significant decrease (Fig. 3G). Finally, we performed Trap staining comparisons using the blank osteoclast induction group, FNC group, and FMgll group of raw264.7 cells, which indicated that the high-fat group could induce osteoclast activation, and silencing the mgll gene could partially attenuate this process (Fig. 3D).

### The degree of liver damage in mice with liver fibrosis was significantly alleviated after treatment with MJN110

**Figure 4:**
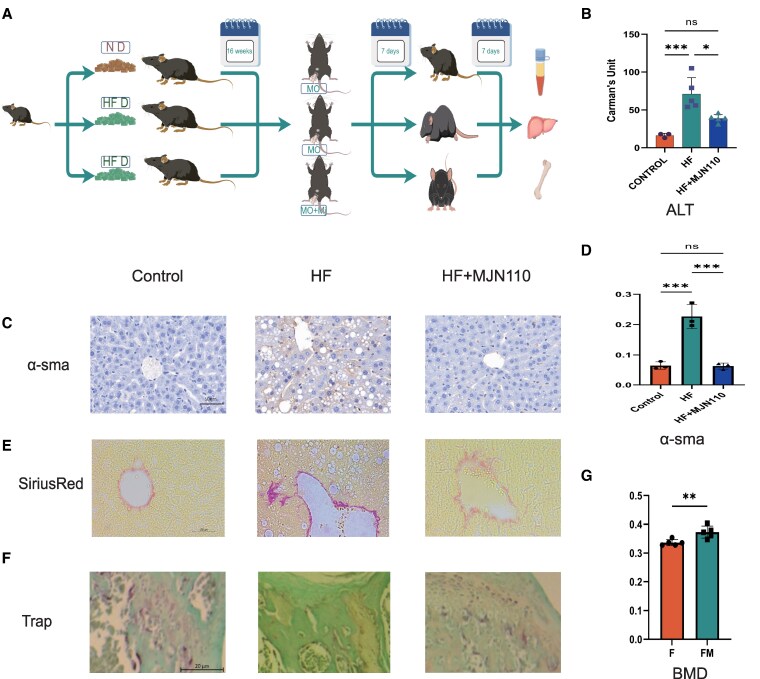
Experimental process of C57BL/6J mice (control, blank control group; HF, high-fat group; HF + MJN110,high-fat + MJN110 group.). A. Schematic diagram of the in vivo experimental process (MO = mineral oil, MO + M = mineral oil + MJN110). B. Determination of serum ALT levels representing the degree of liver damage. C. Representative images of a-sma immunostaining representing the degree of mouse liver fibrosis. D. Statistical analysis of the proportion of positive areas of α-smooth muscle actin (α-sma) in α-sma immunostaining representing the degree of liver fibrosis. E. Representative images of Sirius red staining representing the degree of mouse liver fibrosis. F. Representative images of Trap staining of the lower femur of mice. G. Statistical analysis of bone mineral density in mouse femoral trabecular. *P < .05, **P < .01, ***P < .001; ns, no significant difference. One-way ANOVA followed by Tukey's multiple comparison test (B, C), *t*-test (G). Data are expressed as mean ± SEM.

To elucidate the impact of MJN110 on hepatic function in mice, we performed subsequent experiments as outlined in Fig. 4A. To assess the potential of MJN110 to ameliorate liver function in a murine model of liver fibrosis following intraperitoneal administration, we collected serum samples and quantified alanine aminotransferase (ALT) levels. This was achieved using an ELISA reader, in accordance with the manufacturer's protocol, and ALT concentrations were determined via a standard curve. Our findings revealed that the high-fat diet (HF) group exhibited significantly elevated serum ALT levels compared to the control (C) group. Notably, following a high-fat diet and treatment with the MAGL inhibitor MJN110, ALT levels in the HF + MJN110 group were significantly reduced, reaching levels comparable to the control group (Fig. 4B).

To ascertain whether our experimental paradigm successfully induced the intended degree of hepatic fibrosis in the murine model and to elucidate the impact of MJN110 on liver architecture, we conducted α-smooth muscle actin (α-sma) immunopathological section analysis. α-sma, a well-established marker of hepatic stellate cell (HSC) activation, is frequently employed in liver disease research and serves as an indicator of liver fibrosis severity. The α-sma-positive area in immunohistochemical pathological sections is readily quantifiable using ImageJ software. Consequently, we selected this metric to assess the extent of liver fibrosis in our experimental pathological findings. The results demonstrated a significant increase in the proportion of α-sma-positive areas in the HF group compared to the C group, whereas the HF + MJN110 group exhibited a significant reduction in positive area relative to the HF group (Fig. 4C and D).

Sirius Red, a strongly acidic dye, exhibits a high affinity for the alkaline moieties within collagen molecules, resulting in the characteristic red staining of collagen fibers. This technique serves as a valuable pathological staining method for assessing the extent of hepatic fibrosis in experimental murine models. We performed Sirius Red staining on liver sections obtained from mice. Histopathological analysis revealed an expansion and irregularity in the Sirius Red-positive staining area within the liver of the HF group, whereas the HF + MJN110 group exhibited a reduced, albeit irregular, staining pattern (Fig. 4E).

### Mice with liver fibrosis showed enhanced osteoclast activation, which was reversed by MJN110 treatment

Given that this study primarily investigates the impact of MAGL on murine osteoclasts, we subsequently conducted Trap staining on the distal femur sections of mice. The findings revealed an increase in the blue-green area indicative of Trap-positive staining in the HF group, suggesting heightened osteoclast activation. This observation implies that the distal femur is undergoing osteoporotic changes, characterized by bone destruction. In contrast, the HF + MJN110 group exhibited a reduction in the blue-green area associated with Trap-positive staining, which reverted to levels comparable to the control group. This suggests diminished activation of osteoclasts, indicating a trend toward the amelioration of osteoporosis in the distal femurs (Fig. 4F).

### MJN110 treatment attenuated osteoporosis in mice with hepatic fibrosis

In this study, we induced liver fibrosis in mice via a high-fat diet, which resulted in reduced femoral trabecular bone mineral density, as detailed in Fig. 4A. Micro-CT analysis of the distal femoral trabecular was performed to statistically analyze the results. We observed that the MJN110-treated FM group exhibited a significant recovery in bone mineral density compared to the F group, which received only the solvent control (Fig. 4G). These findings suggest that MJN110 treatment ameliorated the observed osteoporosis.

## Discussion

Research indicates that almost all chronic liver diseases are accompanied by changes in bone metabolism, characterized by decreased bone mineral density and structural deterioration, ultimately increasing the risk of fragility fractures [3]. The majority of chronic liver diseases in the literature are characterized by liver fibrosis or cirrhosis, and the specific mechanisms underlying their pathogenesis have been scarcely investigated. Our study demonstrates that MJN110 attenuates osteoclast activation and promotes the recovery of the femoral germinal layer by inhibiting the hepatic synthesis and secretion of MAGL. This research suggests that MJN110 not only ameliorates osteoporosis induced by osteoclast activation but also restores liver function. Mechanistically, building upon prior research on MAGL-mediated inflammatory responses in the liver, our study further revealed that MJN110 inhibits the synthesis of CB1R, a factor implicated in the hepatic fibrosis process, thereby diminishing the regulatory process of osteoclast activation, which is linked to the aforementioned inflammation and oxidative stress pathways. Consequently, we propose a novel therapeutic strategy for hepatic osteoporosis, involving the inhibition of hepatic MAGL synthesis to suppress liver inflammation and fibrosis, ultimately improving hepatic osteoporosis.

Our investigation enhanced the comprehension of hepatic osteoporosis stemming from high-fat diet-induced liver fibrosis. Previous literature revealed that, despite the recognized association between chronic liver disease and osteoporosis, a high-caliber research in this domain is limited. This scarcity likely arises from the challenges associated with primary hepatocyte extraction, hepatocyte fragility, and the difficulties in cell passage, compounded by limited interdisciplinary knowledge. Some investigators have explored the transplantation of human exfoliated deciduous tooth stem cell-derived hepatocyte-like cells (SHED-Heps) to address bone loss in chronic liver fibrosis, achieving some advancements [18]. Other studies have focused on lipid metabolism, demonstrating that PP2Aca inhibition can induce LCAT expression. LCAT not only mitigates bone loss but also ameliorates chronic liver damage, improving liver fibrosis and function by promoting reverse cholesterol transport in HOD [19]. Furthermore, researchers have analyzed clinical indicators from patients with nonalcoholic fatty liver disease (NAFLD) or metabolic dysfunction-associated fatty liver disease (MAFLD) [7, 20], concluding that a liver–bone axis connection, leading to osteoporosis, is only established during the liver fibrosis stage. These findings collectively suggest that hepatic osteoporosis manifests only when liver function decompensation progresses to liver fibrosis or cirrhosis. Consequently, our experimental model was designed to induce liver fibrosis through a high-fat diet. Our research findings confirm that severe liver damage in the fibrosis stage triggers bone resorption via osteoclast activation. The application of MJN110, which inhibits the MAGL synthesis-mediated liver fibrosis process, attenuates mouse osteoclast activation, thereby decelerating osteoporosis progression. This further substantiates the theoretical framework for the diagnosis and treatment of hepatic osteoporosis.

Hepatic MAGL not only contributes to hepatocyte injury and promotes hepatic inflammation and fibrosis but also exacerbates these processes by stimulating Kupffer cells. The MAGL inhibitor MJN110 elicits anti-inflammatory and antifibrotic effects by inducing autophagy in Kupffer cells15. While MAGL has been less studied in the liver, it has been extensively investigated in brain tissue. Studies have demonstrated that genetic inactivation of MAGL results in a significant reduction in brain 2-AG hydrolysis activity, a 10-fold increase in brain 2-AG levels, and a corresponding decrease in arachidonic acid (AA) levels. MAGL inhibition can elevate brain 2-AG levels, which, coupled with antianxiety, antidepressant, and antinociceptive effects mediated through CB1 receptors, may offer therapeutic potential for neuropathic pain and neurodegenerative diseases associated with neuroinflammation (eg, Alzheimer's disease, Parkinson's disease, or multiple sclerosis) [21]. To eliminate the influence of other organs and systemic factors, given MAGL's widespread distribution, we employed hepatocyte-specific mgll gene silencing (the gene encoding the MAGL protein) for subsequent validation. This validation confirmed that osteoclasts cocultured with the supernatant of hepatocytes with silenced mgll exhibited altered activation, consistent with prior in vivo findings.

2-AG, a more potent agonist of cannabinoid receptors (including CB1R and CB2R) than N-arachidonic acid ethanolamine (arachidonic acid ethanolamine, AEA), can lead to reduced CB1R agonist sensitivity with sustained elevation. Both 2-AG and AEA, as endogenous cannabinoids, are found in extracellular vesicles, suggesting their involvement in release and intercellular transport [21]. Further investigation is warranted to determine whether CB1R, which exhibits increased expression under pathological conditions despite low expression in the normal liver, and MAGL, which degrades its activator 2-AG, are directly linked in the pathogenesis of liver damage.

Currently, 3 prevalent methodologies are employed for modeling hepatic fibrosis in animal models: high-fat diet (HFD) administration, intraperitoneal carbon tetrachloride (CCl4) injection, and bile duct ligation (BDL). While the majority of prior investigations have utilized CCl4 injections, HFD-induced liver fibrosis, though less frequently reported, may more closely mimic the clinical scenario. This study adopted the HFD approach. Furthermore, the role of monoacylglycerol lipase (MAGL) in liver fibrosis has been scarcely investigated, and no studies have elucidated the liver–bone axis crosstalk. Our findings demonstrate that MAGL not only exacerbates liver fibrosis but also that MAGL-mediated hepatocyte damage stimulates osteoclasts activation, leading to bone degradation in mice. The MAGL inhibitor, MJN110, ameliorated this pathological process. Given the ubiquitous cellular distribution of MAGL, the development of hepatocyte-specific MAGL inhibitors remains challenging. However, it is hypothesized that such a drug could offer therapeutic benefits for both liver fibrosis and hepatic osteoporosis.

### Study limitations

This study is subject to certain limitations. Firstly, the etiology of liver fibrosis is multifactorial; therefore, the HFD model employed in this study does not encompass the viral, drug-induced, and cholestatic etiologies observed clinically. Secondly, although primary mouse hepatocytes were initially isolated, purification and propagation proved challenging due to contamination and resource constraints, leading to the abandonment of primary cell-based experiments and mechanistic investigations involving osteoblasts. Finally, a larger sample size may be required to mitigate potential bias.

## Data Availability

The data that support the findings of this study are available from the corresponding author upon reasonable request. The data are not publicly available due to privacy restrictions. Requests should be directed to Liangliang Wang, email: liangliangwang@njmu.edu.cn.
